# The Effects of Eccentric Web Openings on the Compressive Performance of Pultruded GFRP Boxes Wrapped with GFRP and CFRP Sheets

**DOI:** 10.3390/polym14214567

**Published:** 2022-10-27

**Authors:** Emrah Madenci, Yasin Onuralp Özkılıç, Ceyhun Aksoylu, Alexander Safonov

**Affiliations:** 1 Department of Civil Engineering, Necmettin Erbakan University, Konya 42140, Turkey; 2 Department of Civil Engineering, Konya Technical University, Konya 42130, Turkey; 3 Center for Materials Technologies, Skolkovo Institute of Science and Technology, 121205 Moscow, Russia

**Keywords:** web openings, composite materials, EBR method, fiber-reinforced materials, compressive, pultruded GFRP, FRP wrapping

## Abstract

Pultruded fiber-reinforced polymer (PFRP) profiles have started to find widespread use in the structure industry. The position of the web openings on these elements, which are especially exposed to axial pressure force, causes a change in the behavior. In this study, a total of 21 pultruded box profiles were tested under vertical loads and some of them were strengthened with carbon-FRP (CFRP) and glass-FRP (GFRP). The location, number and reinforcement type of the web openings on the profiles were taken into account as parameters. As a result of the axial test, it was understood that when a hole with a certain diameter is to be drilled on the profile, its position and number are very important. The height-centered openings in the middle of the web had the least effect on the reduction in the load-carrying capacity and the stability of the profile. In addition, it has been determined that the web openings away from the center and especially the eccentric opening significantly reduces the load carrying capacity. Furthermore, when double holes were drilled close to each other, a significant decrease in the capacity was observed and strengthening had the least effect on these specimens. It was also determined that the specimens reinforced with carbon FRP contribute more to the load-carrying capacity than GFRP.

## 1. Introduction

Pultruded fiber-reinforced polymer (PFRP) material is used by many engineers, technicians and architects as an alternative to concrete and steel materials [1,2,3,4,5]. Recently, there has been great interest in the applications of PFRP profiles. Because of their many outstanding characteristics such as flexibility, high strength, and chemical resistance, PFRPs have been used widely in many fields of building and infrastructure structures [6]. The PFRP materials that were used in bridge and building structures remained effective up to 17 years after installation [7]. PFRP increases the strength and stiffness while reducing structure mass [8]. Numerous developing applications of composite materials have been found in recent years as a result of utilizing glass, carbon, boron, and aramid fibers along with ceramic and metal matrixes [9]. A number of investigations on experimental studies of PFRP composite structures can be found in the literature [10,11,12,13,14,15]. Madenci et al. [16] experimentally and theoretically investigated the flexure performances of pultruded glass FRP (PGFRP) composite beams. Theoretical approaches for the flexural analysis of PGFRP composite beams were developed with the help of variational methods. An exact analytical solution based on first-order shear deformation plate theory was used for the solution of stability and vibration problems by Madenci et al. [17]. The virtual displacement principle was utilized herein to derive governing differential equations. the effective material properties of PGFRP composites were obtained using the mixture rule model. Correia [18] presented experimental and numerical studies about the structural behavior of a pultruded glass PGFRP beam and a hybrid, namely carbon and glass FRP beam, respectively. Hai et al. [19] experimentally investigated the flexural behavior of hybrid composite beams comprising different carbon fiber contents.

Among the FRP manufacturing technologies, pultrusion is a sequential process that aims to produce FRP elongated members of a constant cross-section using continuous fibers that are soaked in resin and heated to cure them [20]. In general, the fibrous architecture of PFRPs is composed of several different reinforcement structures designed to respond to any case-specific necessities. Typically, the core is reinforced by unidirectional fibers, which provide the longitudinal resistance and stiffness of the profile. Unidirectional or multiaxial fabrics, made of well-ordered fiber mats, are employed to enhance the transversal or off-axis properties in a specific direction [21].

In comparison with isotropic materials, composites have a wider range of parameters influencing the structural behavior. Ply orientations, fiber volume fraction, number of layers, stacking sequence, the material of fibers and matrix and the thickness of layers are examples of these effective parameters which can act as design variables in optimization problems [22,23,24]. Saghir et al. [25] presented an experimental program to better understand the individual and consolidated effect of the constituents upon mechanical strengths: axial tensile and hoop tensile strengths of GFRP mortar pipes and to apply this improved understanding towards the formulation of the semi-empirical prediction models. Tashnizi et al. [26] reported on research that seeks to design laminated cylindrical CFRP composite pipes with an optimal winding angle (*ϕ*) to boost their mechanical strength against patch loading. To observe the sole effect of the winding angle on the pipe’s mechanical strength, the stacking sequence of the composite laminates ([*ϕ*/-*ϕ*/*ϕ*/-*ϕ*/*ϕ*]) was kept constant. Gohari et al. [27] presented a systematic approach towards the localized failure inspection of internally pressurized laminated ellipsoidal woven composite domes. The domes were made of thin glass fiber-reinforced polymer (GFRP) woven composite layups [0,0,0], [0,30,0], [0,45,0], and [0,75,0]. The current design standards and manuals are still basic and only contain conservative formulas for the design with no considerations for the interactions between the design parameters. This lack of knowledge discourages design engineers and contractors from heavily relying on these profiles in infrastructure applications due to uncertainty and overdesign. The structural design of FRP composites requires more specifications compared to isotropic materials since the layup and geometric parameters have to be assigned for composites while only the dimensions are to be determined for isotropic material [28].

In structural frameworks made with traditional materials such as reinforced concrete and steel, web openings are often applied in beams, columns, and floors for passage channels to house vital services which may include air conditioning, electricity, telephone, water supply, and a system network. These openings can be rectangular, circular, trapezoidal, triangular, diamond-shaped, and sometimes irregular in shape and located in the center of the building element, on edge, above the center. Most of the existing studies on the behavior of reinforced concrete (RC) beams with a web opening chose to use the FRP reinforcement for the strengthening of the opening region [29,30]. FRP was found to be effective for strengthening RC members in structures because of its excellent mechanical properties [31,32,33,34,35,36,37,38,39,40,41,42,43,44,45]. FRP reinforcement schemes can be implemented in different ways. The most used FRP schemes in the literature can be listed as “vertical side bonded FRP sheets/plates or on the two sides of the opening [46,47,48,49]; vertically bonded FRP U-jackets or on the two sides of the opening [50,51]; vertically bonded FRP complete wraps or on the two sides of the opening [52]; diagonal side bonded FRP sheets/plates near the corners of the opening [53]; horizontally bonded FRP sheets/plates on the side surfaces or on the top and bottom surfaces of the beam [54]; diagonal near-surface mounted FRP bars at the opening corners”. Aksoylu et al. [55] experimentally examined the effect of web openings on the response of pultruded fiber-reinforced polymer (PFRP) composite profiles under compressive loads. A number of specimens have been processed to examine the behavior of PFRP profiles with centered web openings. The effects of the size of the web opening and the FRP-strengthening scheme on the structural performance of PFRP profiles with FRP-strengthened web openings have been thoroughly analyzed and discussed.

The aim of this research was to examine the effect of eccentric openings on the compressive behavior of the pultruded profiles. A total of 21 pultruded box profiles with reinforced FRP were prepared and tested under compressive loads. The opening location, number of holes and strengthening scheme are considered parameters of this study.

## 2. Experimental Program

In this study, the compressive behavior of the pultruded box with eccentric openings was examined through the experimental study. The opening locations and number of circular holes were considered as primary variables while the strengthening of the openings with FRPs was selected as the secondary variable. The size of the pultruded profiles used in the experiments is 75 mm × 75 mm × 6 mm and the length of these specimens is 150 mm. These profiles were produced by FİBERR Fiber Reinforced Composites Company, Manisa, Turkey. The mechanical properties of these pultruded GFRP profiles are given in Table 1.

**Figure 1:**
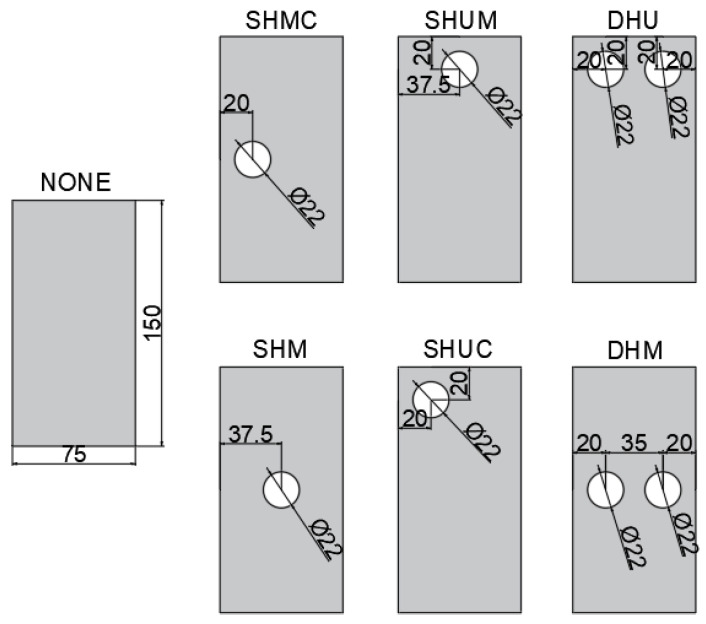
Specimens with same opening size with different location, dimensions are mm.

Hole diameters were kept constant at 22 mm. Reference specimens without holes were also considered. The openings were considered at different locations and two holes were also drilled in some specimens to investigate the effects of the number of holes on the capacity. The specimens were coded as no holes (None); single-hole center (SHC); single-hole middle corner (SHMC); single-hole upper corner (SHUC); single-hole upper middle (SHUM); double-hole middle (DHM); and double-hole upper (DHU). The specimens with openings are shown in Figure 1. Vertical loads were applied to the samples in the direction shown in Figure 1. Furthermore, the specimens were strengthened with two types of composites: single-layer 800 gr/m^2^ 0° CFRP and 1200 gr/m^2^ 0° GFRP. The fiber direction is in the direction of the load. P represents no strengthening, C represents CFRP strengthening, and G represents GFRP strengthening.

For FRP applications, F-1564 resin and F-3486-3487 hardener were used. Based on the manufacturer’s data, the resin and hardener mixing ratio was taken as 100/34 (by weight). The resin and hardener were carefully applied to gain bonding between the pultruded box and FRP fabric. The material properties of FRP fabrics are given in Table 2. All specimens were fully wrapped.

**Figure 2:**
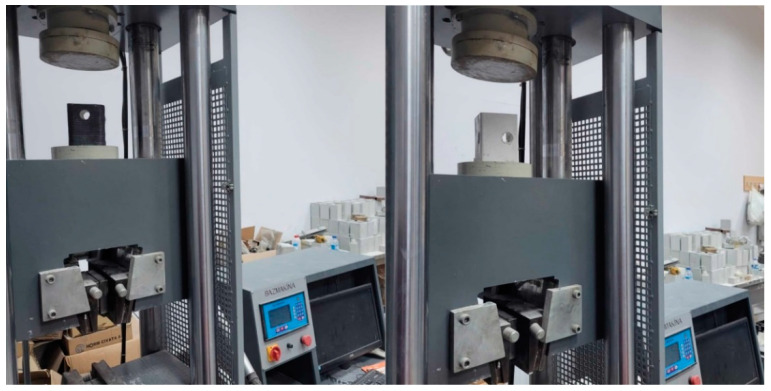
Test setup.

The specimens were tested under compressive loading. The test setup with a capacity of 600 kN shown in Figure 2 was utilized to perform the tests. The test setup has a servo-controlled hydraulic actuator which has a loadcell with a capacity of 600 kN and a displacement sensor. The displacements and loads were automatically recorded during the experiments. The specimens were loaded at a speed of 2 kN/s.

## 3. Results and Discussion

Furthermore, the effects of hole location and the number of holes were studied under three different wrapping conditions. Table 3 depicts the load capacities of these specimens. In here, SHM represents the specimens with openings located at the center of mid-height and width. SHMC represents the specimens with an opening located 20 mm away from the corner at mid height. SHUC represents the specimens with the opening located 20 mm away from the corner and 20 mm away from the upper edge. The SHUM represents the specimens with an opening located away from the upper edge at the center. DHM represents the specimens with two openings located 20 mm away from each corner at mid-height. DHU represents the specimens with two openings located 20 mm away from each corner and 20 mm away from the upper edge.

Damage analyses of PGFRP box-section profiles and load–displacement relations are summarized as follows. The location of the hole characterizes damage behavior and load–displacement curves.

**Figure 3:**
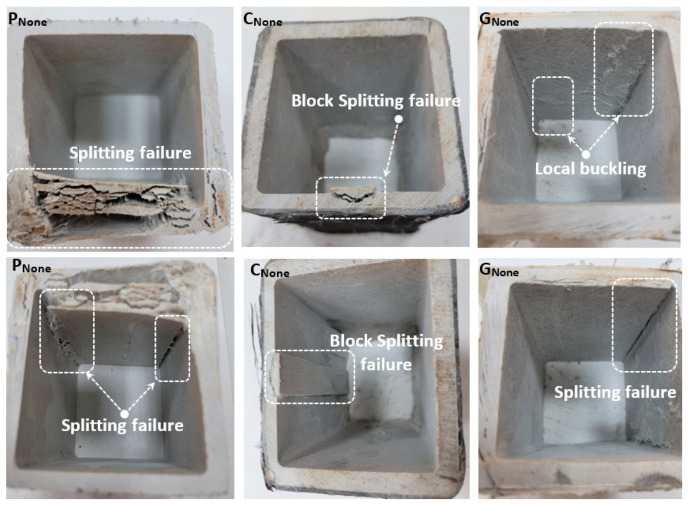
Damage analyses of P_None_, C_None_, G_None_.

Compared to P_None_, the load-carrying capacity of the carbon and glass-wrapped C_None_ and G_None_ specimens increased by 3.1% and 1.7%, respectively, when comparing the specimens with no holes drilled first. However, the initial stiffness values increased due to the winding effect. This situation is understood by the increase in the angle made with the horizontal in the load-displacement curve. In addition, comparative damage analyses are shown in Figure 3. When Figure 3 is examined, crushing and splitting failure damage was observed in the profile cross-section due to the pressure effect in the P_None_ specimen. In addition, splitting failure occurred at the corner points along the profile height, and the experiment was terminated. As a result of the reinforcement of the P_None_ specimen with carbon and glass fiber, it was determined that there were significant differences in the damage behavior. First, no splitting damage was observed in the profile cross-section. In addition, as a result of reinforcement with carbon fiber, splitting failure damage at corner points is prevented. However, the damage type caused a block splitting failure in the middle of an edge. Glass fiber reinforcement partially reduced the splitting damage at the corner points. However, local buckling failure has been observed here. As a result of the damage analysis, the applied carbon and glass fiber reinforcements did not show a significant increase in the load-carrying capacity of the unperforated specimen, but changing the final damage type was an indicator of the wrapping efficiency.

**Figure 4:**
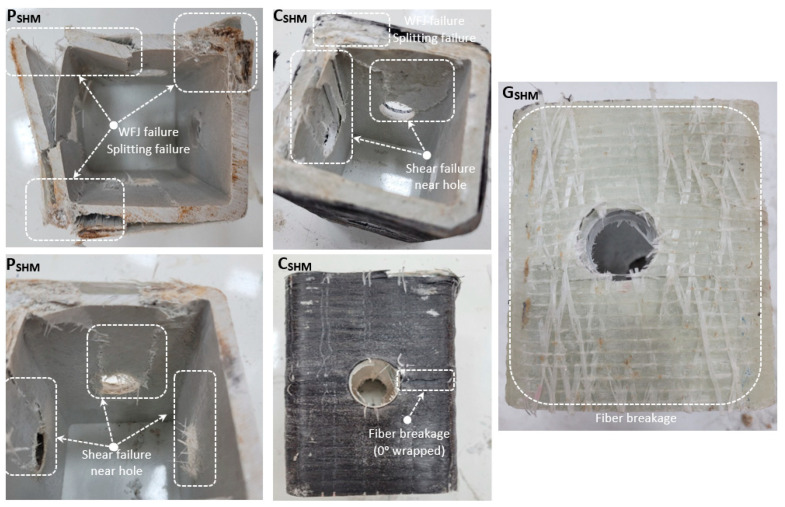
Damage analyses of P_SHM_, C_SHM_, G_SHM_.

Significant differences occurred in the load-carrying capacity and damage analyses of the specimens called SHM, which opened with a diameter of 22 mm in the middle of the four sides of the PGFRP profile (in Figure 4). According to P_SHM_, there was an increase of 3.0% and 1.9% in the load carrying capacity of C_SHM_ and G_SHM_, respectively. Although reinforcement with the carbon fiber provides an increase in the load-carrying capacity compared to glass fiber, the increase rate is at a very low level compared to P_SHM_. As the load increased in P_SHM_, shear damages occurred around the hole, crushing in the pressure region and splitting damages occurred in the web flange junction (WFJ) region, and the experiment was terminated. At the end of the experiment, the reinforcements applied with carbon and glass prevented the profile from separating from the corner points. However, in both reinforcement applications, shear damage around the hole and fiber breakage were observed on the profile surface. The fact that the profile remains, as a whole, at the end of the test and no debonding damage is observed indicates that the selected fiber materials have sufficient strength and that the winding is effective.

**Figure 5:**
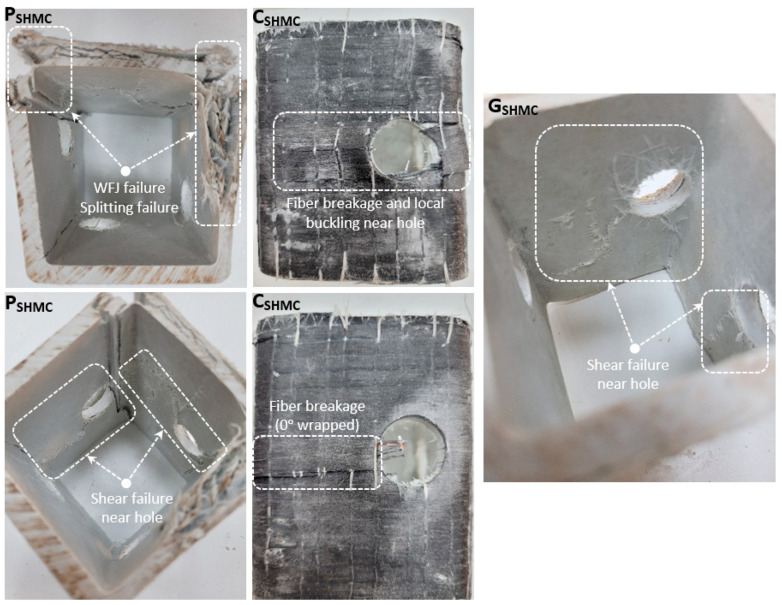
Damage analyses of P_SHMC_, C_SHMC_, G_SHMC_.

The 22 mm hole diameter drilled into the SHMC sample was drilled in the middle of the height and 20 mm inside from the corner (in Figure 5). In addition, the SHMC sample was reinforced with CFRP and GFRP. Increases of 10.9% and 8.5% were observed in the load-carrying capacity of the reinforced samples (C_SHMC_ and G_SHMC_) compared to P_SHMC_, respectively. The load-carrying capacity of PSHMC decreased by 13.9% compared to P_SHM_, while the reinforced C_SHMC_ and G_SHMC_ decreased by 7.3% and 8.3% compared to C_SHM_ and G_SHM_, respectively. Strengthening the hole drilled in the S_HMC_ sample with CFRP and GFRP both changed the damage type and limited the decrease in load-carrying capacity. When the specimens whose damage analysis is given in Figure 5 are examined, a splitting failure was observed in the WFJs in P_SHMC_ due to the pressure effect. In addition, shear damage occurred around the hole. Here, the shifting of the hole on the specimen to the edge caused a decrease in the load-carrying capacity and a faster collapse. WFJ damage was not seen in both C_SHMC_ and G_SHMC_, and WFJ damage, especially in the corner areas, was prevented. Local buckling damage was observed around the hole in C_SHMC_ and G_SHMC_, while shear damage was observed around the hole. The fact that the drilled hole is close to the edge caused the applied load to be eccentric and the damages to occur, especially in the area close to the hole. This prevented the formation of WFJ damage in the upper parts of the strengthened specimens. Because of fiber breakage, shear failure and local buckling damage around the hole, which is the weakest link, this accelerated the end of the experiment.

**Figure 6:**
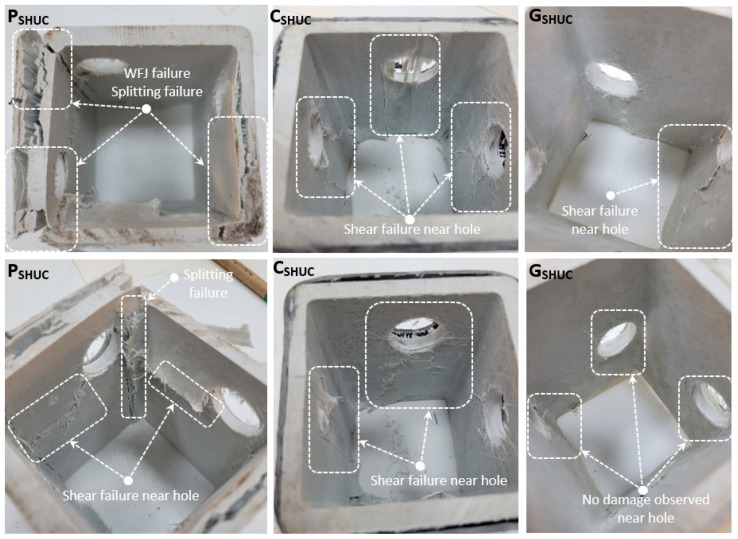
Damage analyses of P_SHUC_, C_SHUC_, G_SHUC_.

The hole location in the SHUC sample is similar to the SHMC and was created 20 mm from the bottom and edge (in Figure 6). This led to a reduction in load-carrying capacity. According to P_SHUC_, there was a 10.9% and 7.2% increase in payload capacity at C_SHUC_ and GSHUC, respectively. The load-bearing capacity of P_SHUC_ decreased by 19.1% compared to P_SHM_, while the fortified C_SHUC_ and G_SHUC_ decreased by 12.8% and 14.9% compared to C_SHM_ and G_SHM_, respectively. This showed that the web opening eccentrically away from the center reaches less load-carrying capacity. Splitting damages were observed in the WFJ part of the P_SHUC_, which was considered as a reference (Figure 6). In addition, shear damage around the hole and splitting failure occurred at the corners along the profile height. The fact that the web opening on the specimen was at the bottom and close to the edge caused a further decrease in the load-carrying capacity and a faster collapse compared to P_SHM_. WFJ damage was not seen in both reinforced C_SHUC_ and G_SHUC_, and WFJ damage, especially in the corner areas, was prevented. In addition, the specimens reinforced with carbon had more shear damage around the hole, while the specimens reinforced with GFRP had shear damage around only one hole. The fact that the web opening was at the bottom and close to the edge caused the damage to occur, especially in the area close to the hole. This prevented the formation of WFJ damage in the upper caps of the strengthened specimens. Because the shear failure damage around the hole, which is the weakest link, caused the experiment to be terminated.

**Figure 7:**
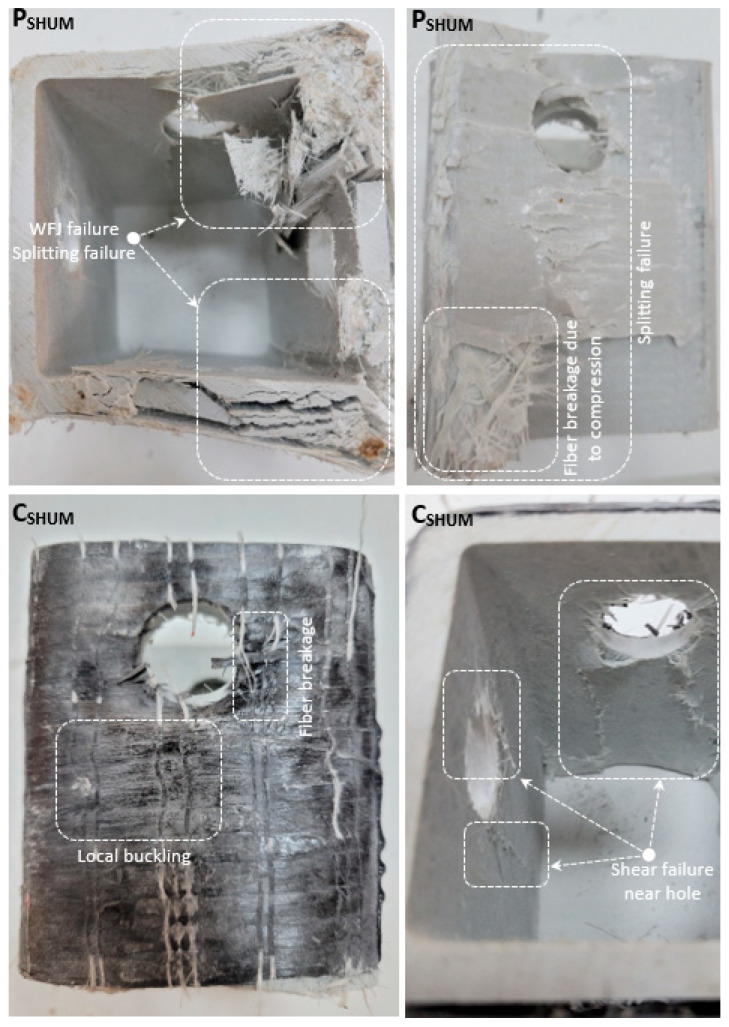
Damage analyses of P_SHUM_, C_SHUM_, G_SHUM_.

The load-carrying capacity of the unreinforced P_SHUM_ specimen increased by 16.1% and 9.2%, respectively, compared to P_SHUC_ and P_SHMC_, and decreased by 6% and 13.5%, respectively, compared to P_SHM_ and P_None_. This shows that the hole diameters opening in the center of the profile contribute more to the load-carrying capacity. Increases of 4.2% and 2.5%, respectively, were observed in the load-carrying capacity of the reinforced specimens (C_SHUM_ and G_SHUM_) compared to P_SHUM_. In addition, the rate of increase in specimens with an eccentrically web opening diameter is similar. This shows that the increase in load-carrying capacity is limited by strengthening the web opening in the center of the specimens. Therefore, it is understood that reinforcement materials contribute to carrying more load in specimens with eccentric placement. The rate of increase in C_SHUM_ and G_SHUM_ and the rate of increase in C_SHM_ and G_SHM_ (3% and 1.9%, respectively) are approximately similar. The load-carrying capacity of C_SHUM_ and G_SHUM_ decreased by 4.9% and 12.6%, respectively, compared to C_SHM_ and G_SHM_, while it decreased by 5.4% and 12.8% compared to C_None_ and G_None_. This shows that the load-carrying capacity is further increased by strengthening the holes in the middle of the specimen’s height. In the P_SHUM_ specimen, splitting damage occurred in the WFJ part with the effect of pressure (Figure 7).

In addition, splitting failure was observed in the corner part along the profile height. Particularly, shear damage occurred around the hole, which is the weakest link, causing a sudden decrease in the load-carrying capacity. However, this damage occurred at a higher load value than P_SHUC_, depending on the hole position. Both reinforced C_SHUM_ and G_SHUM_ have no WFJ damage, and WFJ damage, especially in corner areas, is blocked by reinforcement materials. In addition, shear damage occurred around the hole of the specimens reinforced with CFRP and GFRP, however, the progression of these damages was limited. As a result, the reinforced specimens had a more stable appearance at the end of the experiment, and the absence of debonding damage showed that the selected fiber materials were suitable for reinforcement.

**Figure 8:**
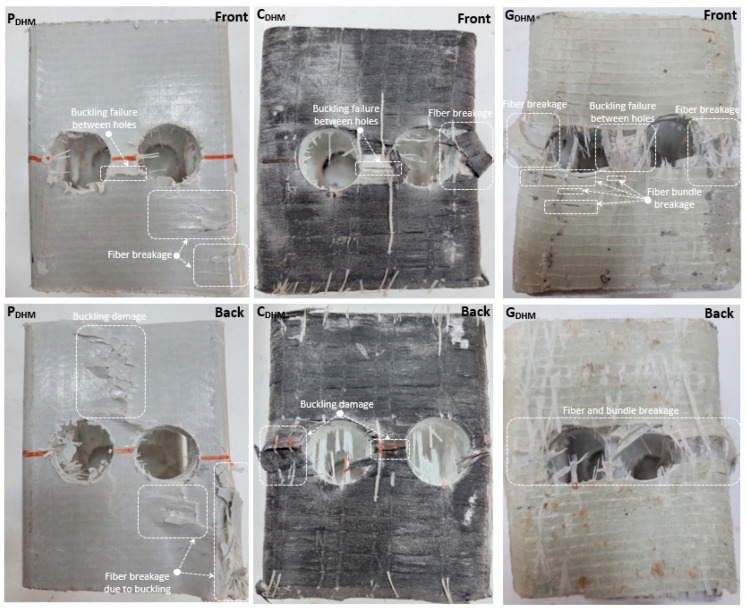
Damage analyses of P_DHM_, C_DHM_, and G_DHM_.

Since the number of web openings in the DHM specimen is two on one side, the load-carrying capacity is significantly less than the specimens with a single hole diameter. While the load-carrying capacity of P_DHM_ decreased by 53.5% compared to P_None_, the decrease rate of C_DHM_ and G_DHM_ compared to C_None_ and G_None_ specimen was 49.6% and 52.9%, respectively. The increase rate of the strengthened C_DHM_ and G_DHM_ compared to P_DHM_ was 12% and 3.1%, respectively. This shows that the reinforcement made with carbon fiber contributes more to the load-carrying capacity than glass fiber. The load-carrying capacity of the P_DHM_ specimen compared to P_SHMC_ decreased by 41.4%, while the load-carrying capacity of C_DHM_ and G_DHM_ compared to C_SHMC_ and G_SHMC_ decreased by 40.8% and 44.3%, respectively. In addition, it is understood that the two web openings in the profile considerably reduced the load-carrying capacity and that these specimens cannot be effective even if they are strengthened. As can be seen in Figure 8, a buckling failure occurred between the web openings in P_DHM_, C_DHM,_ and G_DHM_ specimens. This is due to the small distance between the holes. In addition, fiber breakage damage was observed in the corner region of P_DHM_ with the effect of pressure, while this damage was prevented in the strengthened C_DHM_ and G_DHM_ specimens. Unlike C_DHM_, fiber bundle breakage damage was also observed in the G_DHM_ specimen. Finally, it was understood from the ruptured fiber materials that the damages were concentrated around the hole, which is the most critical area in the strengthened specimens, and that the fiber materials were forced to prevent this damage.

**Figure 9:**
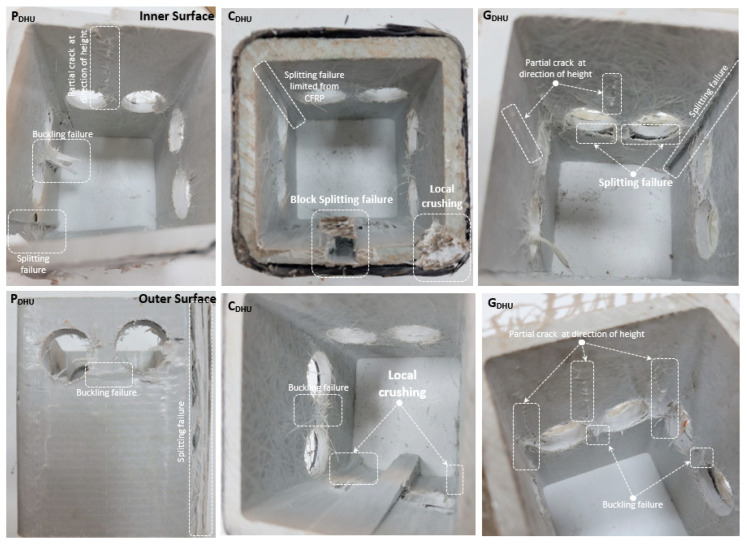
Damage analyses of P_DHU_, C_DHU_, and G_DHU_.

While the load-carrying capacity of the P_DHU_ specimens decreased by 14% compared to P_DHM_, it decreased by 60.1% compared to P_None_. The sample with the least load-bearing capacity among the non-reinforced specimens was obtained to be P_DHU_. This shows that the load-carrying capacity can be significantly reduced according to the number of holes and hole location, and the number and location of the web openings are very important. The load-carrying capacity of the reinforced C_DHU_ and G_DHU_ specimens increased by 20.1% and 10.9% compared to P_DHU_. In addition, the reduction in load-carrying capacity of C_DHU_ and G_DHU_ compared to the C_None_ and G_None_ specimens was 53.5% and 56.5%, respectively. Although the strengthening applications increase the load-carrying capacity, the fact that the load-carrying capacity is quite low compared to the specimen with no holes also shows that the capacity increase after strengthening will not be sufficient for the specimens with such holes. The load-carrying capacity of the P_DHU_ specimen decreased by 14.01%, 53.8%, 46.4%, 49.6%, and 56.6%, respectively, compared to P_DHM_, P_SHUM_, P_SHUC_, P_SHMC,_ and P_SHM_. The decentralization of the hole location greatly reduced the load-carrying capacity. Similarly, the load-carrying capacities of the reinforced CDHU and GDHU specimens were decreased by 7.7–7.4%, 46.8–50.1%, 41.9–44.5%, 45.4–48.5%, and 49.4–52.8% according to CDHM-GDHM, CSHUM-GSHUM, CSHUC-GSHUC, CSHMC-GSHMC, and CSHM-GSHM, respectively. In Figure 9, the damage analyses of P_DHU_, C_DHU_, and G_DHU_ are shown. In the P_DHU_, the damages occurred especially between the two holes. Splitting failure damage was observed in the corner region, as in all other non-reinforced specimens. However, along the specimen height, the cracks starting from the periphery of the hole were elongated and the buckling failure occurred in the region between the two holes. Differences occurred in the damage observed in C_DHU_ and G_DHU_. In C_DHU_, splitting damage was significantly inhibited by CFRP at the corner points, whereas in G_DHU_ splitting damage was quite evident. This led to less load bearing of the GFRP reinforced specimen. In addition, since no splitting damage was observed in C_DHU_, block splitting failure (buckling) occurred in the region between the holes. This situation was not observed in G_DHU_. Splitting damage around the hole and partial crack propagation along the profile height also occurred in G_DHU_. Local crushing damages were observed at the points where the load was applied in all three specimens. The damages caused by the web opening in this specimen are especially concentrated around the hole.

**Figure 10:**
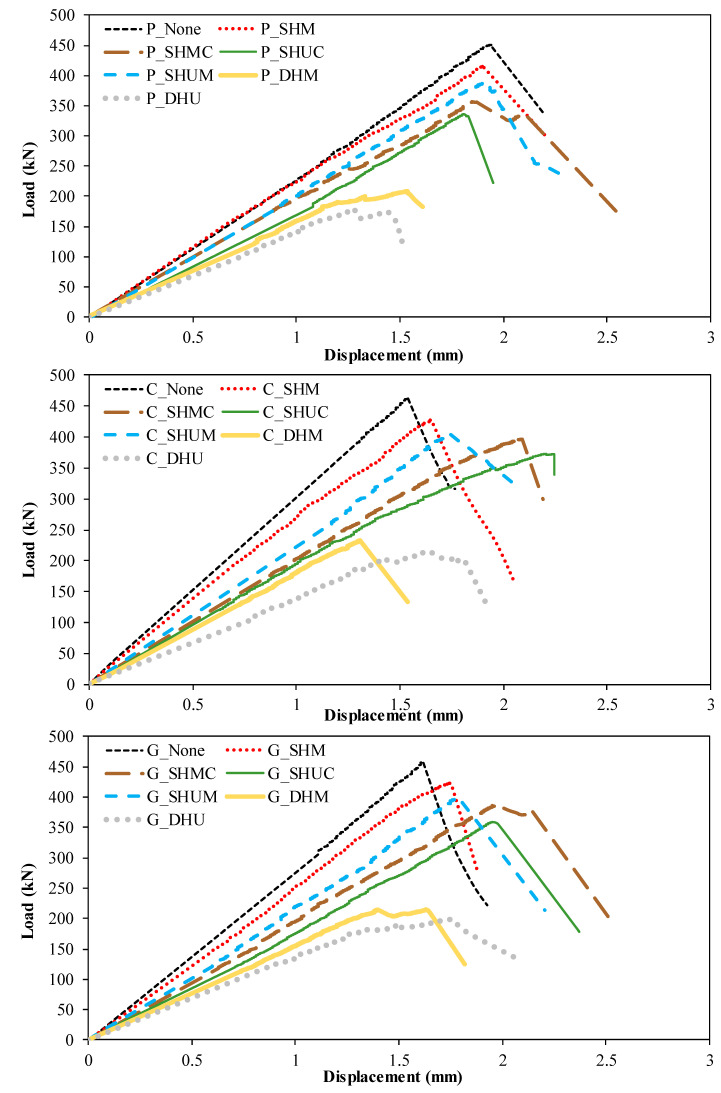
Load–displacement curves of openings with different locations and numbers.

**Figure 11:**
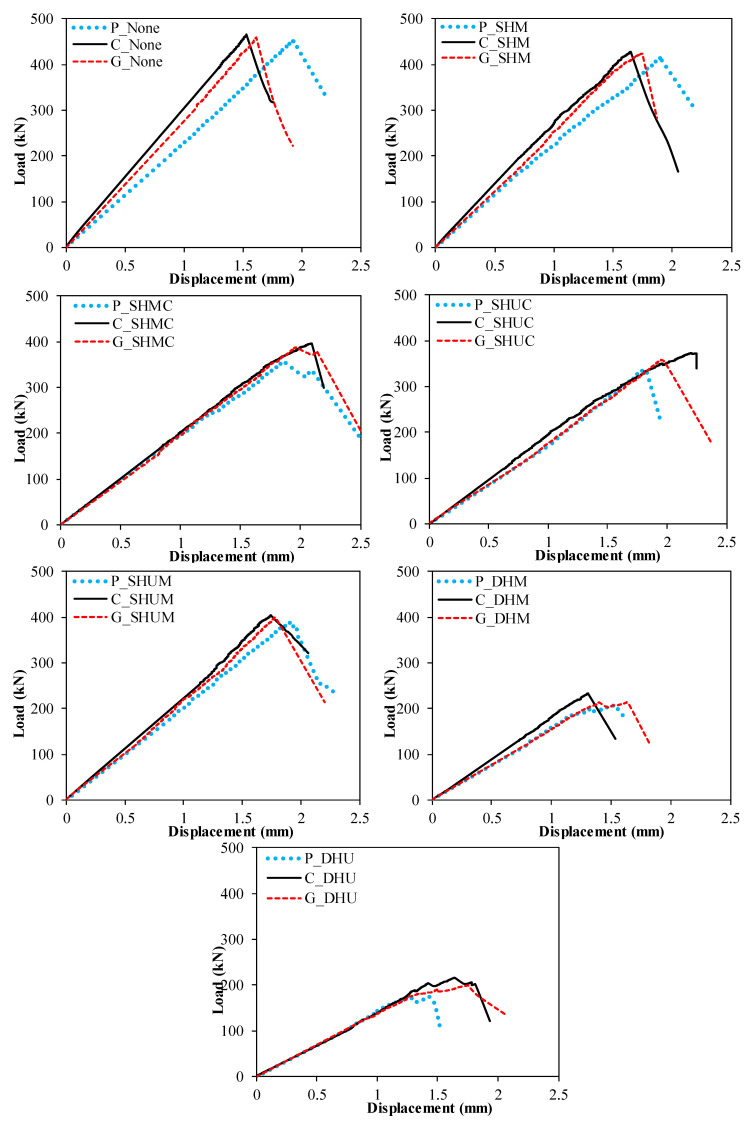
Effects of strengthening on different opening schemes.

The damage analysis and comparison of each specimen were taken into consideration by considering the load-displacement curves shown in Figure 10 and Figure 11. When the specimens represented with P in Figure 11 were compared, the web opening was close to the top of the profile and there were two holes, as a 60.1% decrease in load carrying capacity occurred compared to P_None_. In addition, this ensured that the initial stiffness was minimal. However, according to P_None_, the smallest decrease in load-carrying capacity was observed in the P_SHM_ specimen, where a single hole was opened in the middle at the middle height of the beam, resulting in a 7.9% decrease. This situation occurred similarly in the strengthened specimens.

## 4. Conclusions

Looking at the results, the web openings on the PGFRP profile reduce the bearing capacity of the profile. In order to increase the reduced bearing capacity, GFRP/CFRP fabric wrap was applied to the specimens. With this application, a slight increase in the carrying capacity of the specimens was achieved. However, it was determined that the position of the hole affects the carrying capacity. The experimental testing of the axial load capacity of pultruded box profile, strengthened using CFRP and GFRP composite sheets, allowed us to determine the following conclusions:

(1) The P_None_ specimen has the highest vertical load-carrying capacity among the non-reinforced specimens. The increase in load-carrying capacity compared to other specimens between 8.6% and 150.6% is changing.
(2) The P_DHU_ specimen represented the specimen with the least axial compressive capacity among the non-reinforced specimens. Compared to other specimens, the decrease in capacity varies between 14% and 60.1%.
(3) Among the specimens reinforced with CFRP and GFRP, C_None_ and G_None_ have the highest vertical load-carrying capacity. The increase in capacity of C_None_ and G_None_ specimens compared to other specimens varies between 8.7–115.1% and 8.4–129.8%. From this, it is understood that the specimens reinforced with CFRP contribute more to the load-carrying capacity than GFRP.
(4) As a result of the damage analysis, damages occurred in the form of splitting failure, WFJ failure, buckling around the hole, and shear damages in all specimens symbolized with P. While block splitting failure, fiber breakage, and buckling damages were observed in the specimens strengthened with CFRP, fiber bundle breakage, splitting and buckling damages were observed in the specimens strengthened with GFRP.

As a result of the axial test, it was understood that when a hole with a certain diameter is to be opened on the profile, its position and number are important. It can be stated that the body openings are in the middle and in the middle of the profile height used, leading to an increase in the load-carrying capacity and more stable damages in the perforated specimens. It was determined that the web openings away from the center and especially the eccentric opening significantly reduces the load-carrying capacity. This has led to earlier load damage, especially in the corner areas and around the hole. Therefore, it is recommended that experiments are conducted on pultruded profiles with different widths in order to reach a more precise judgment by creating different hole diameters and locations.

## Figures and Tables

**Table 1:** Mechanical properties of pultruded GFRP.

Property	Mean Value (MPa)
Longitudinal tensile modulus of elasticity	23,000
Transverse tensile modulus of elasticity	7000
Longitudinal tensile strength	240
Transverse tensile strength	50
Longitudinal compressive strength	150
Transverse compressive strength	70
Shear strength	25

**Table 2:** Material properties of FRP fabrics.

CFRP Strip Properties (800 gr/m^2^)	Values
Thickness (mm)	0.85
Tensile strength (GPa)	4.4
Modulus of elasticity (GPa)	235
Rupture strain (%)	1.87
**GFRP strip properties (1200 gr/m^2^)**	**Values**
Thickness (mm)	1.2
Tensile strength (GPa)	3.5
Modulus of elasticity (GPa)	80
Rupture strain (%)	4.37
**Epoxy+Hardener (F-1564+F-3486)**	**Values**
Tensile strength (GPa)	0.055
Modulus of elasticity (GPa)	2.090
Rupture strain (%)	4.06 ± 1.27

**Table 3:** The results of the specimens with different opening locations and number of openings.

Specimen	Hole Size	P (kN)	Decline Ratio %	C (kN)	Decline Ratio %	G (kN)	Decline Ratio %
None	-	450.6	1	464.9	1	458.6	1
SHM	22	414.9	7.9	427.5	8.0	422.8	7.8
SHMC	22	356.9	20.7	396.2	14.7	387.4	15.5
SHUC	22	335.6	25.5	372.4	19.8	359.8	21.5
SHUM	22	389.8	13.4	406.4	12.5	399.8	12.8
DHM	22	209.1	53.5	234.2	49.6	215.6	52.9
DHU	22	179.8	60.1	216.1	53.5	199.5	56.5

## Data Availability

Not applicable.
